# Arbuscular mycorrhizal fungi contribute to reactive oxygen species homeostasis of *Bombax ceiba* L. under drought stress

**DOI:** 10.3389/fmicb.2022.991781

**Published:** 2022-09-20

**Authors:** Zhumei Li, Yanan Zhang, Chao Liu, Yong Gao, Lihong Han, Honglong Chu

**Affiliations:** College of Biological Resource and Food Engineering, Yunnan Engineering Research Center of Fruit Wine, Center for Yunnan Plateau Biological Resources Protection and Utilization, Qujing Normal University, Qujing, China

**Keywords:** drought stress, AMF, reactive oxygen species (ROS), respiratory burst oxidase homologue (Rboh), antioxidant response

## Abstract

Drought stress is one of the major abiotic factors limiting plant growth and causing ecological degradation. The regulation of reactive oxygen species (ROS) generation and ROS scavenging is essential to plant growth under drought stress. To investigate the role of arbuscular mycorrhizal fungi (AMF) on ROS generation and ROS scavenging ability under drought stress in *Bombax ceiba*, the ROS content, the expression levels of respiratory burst oxidase homologue (*Rboh*s), and the antioxidant response were evaluated in AMF and NMF (non-inoculated AMF) plants under drought stress. 14 *BcRboh* genes were identified in the *B. ceiba* genome and divided into five subgroups based on phylogenetic analysis. The effect of AMF on the expression profiles of *BcRboh*s were different under our conditions. AMF mainly downregulated the expression of *Rboh*s (*BcRbohA*, *BcRbohD*, *BcRbohDX2*, *BcRbohE*, *BcRbohFX1*, and *BcRbohI*) in drought-stressed seedlings. For well-water (WW) treatment, AMF slightly upregulated *Rboh*s in seedlings. AMF inoculation decreased the malondialdehyde (MDA) content by 19.11 and 20.85%, decreased the O_2_⋅^–^ production rate by 39.69 and 65.20% and decreased H_2_O_2_ content by 20.06 and 43.21% compared with non-mycorrhizal (NMF) plants under drought stress in root and shoot, respectively. In addition, AMF inoculation increased the non-enzymatic antioxidants glutathione (GSH) and ascorbic acid (AsA) content in roots by 153.52 and 28.18% under drought stress, respectively. The activities of antioxidant enzymes (SOD, PX, CAT, APX, GPX, GR, MDAR, and DHAR) all increased ranging from 19.47 - 131.54% due to AMF inoculation under drought stress. In conclusion, these results reveal that AMF inoculation can maintain ROS homeostasis by mitigating drought-induced ROS burst, via decreasing ROS generation and enhancing ROS scavenging ability of *B. ceiba* seedlings.

## Introduction

Drought stress is one of the most devastating abiotic stressors on plants worldwide (Piao et al., 2010; Guo et al., 2020). Under the relentless ecological degradation of the Anthropocene, we are seeing rising temperatures and decreasing rainfall that suggest drought stress will become increasingly prevalent (Mishra and Singh, 2010; Trenberth et al., 2014). Drought stress triggers a series of detrimental effects on plants, such as membrane system damage, osmotic imbalance, photosynthetic dysfunction, cellular metabolic dysfunction, etc. (Fahad et al., 2017; Hasanuzzaman et al., 2020; Yang et al., 2020a). In plants, drought stress provokes the generation of reactive oxygen species (ROS) and leads to the overaccumulation of ROS. Such as superoxide anion free radical (O_2_^.–^), hydrogen peroxide (H_2_O_2_) and hydroxyl radical (OH⋅), etc. produced in different subcellular compartments (e.g., plasma membranes, chloroplasts, peroxisomes, and mitochondria) (Tiwari et al., 2017; Hasanuzzaman et al., 2021). The production of ROS can happen through a variety of pathways (peroxidases, excited chlorophyll, Glycolate and xanthine oxidase, fatty acid oxidation, and the electron transport chain in photosynthesis and respiration) (Cruz de Carvalho, 2008; Li et al., 2009; Hasanuzzaman et al., 2020). The overaccumulation of these ROS caused by drought stress can induce an ‘oxidative burst,’ thus leading to oxidative injury in plants. Oxidative injury can cause lipid peroxidation in cellular membranes, denature protein, cause nucleic acid damage, increase carbohydrate oxidation, catalyze pigment breakdown, and induce programmed cell death (Raja et al., 2017; Choudhary et al., 2018). This oxidative injury and death can have plant-scale consequences, suggesting that the influence of drought on plant growth and mortality is in large part mediated by ROS overaccumulation (Yang et al., 2020b; Zou et al., 2021). On the other hand, avoiding ROS deficit and maintaining an optimum ROS level plays a vital role in molecular signaling in plant growth, development, adaptation, and in response to various abiotic and biotic stresses (Liu and He, 2016; Mittler, 2017). Therefore, homeostasis between ROS generation and ROS scavenging under stressful environments is a prerequisite of plant success.

*Bombax ceiba* Linn. is a tree in the Malvaceae family, and is commonly called the cotton tree or red silk cotton tree. *B. ceiba* is mainly distributed in tropical and sub-tropical Asia as well as northern Australia (Barwick and Schans, 2004). *B. ceiba* has great economic and ecological importance (Gao et al., 2018) and can be a source of food, medicine, fuel, fiber, fodder, and many cultural goods for natives of many Asian communities (Jain and Verma, 2012; Gao et al., 2018). *B. ceiba* occurs naturally in dry-hot valley areas (Jin et al., 1995; Zhou et al., 2015), and is a reforestation pioneer that survives easily in low-rainfall and well-drained conditions (Zhou et al., 2015). Thus, *B. ceiba* is a candidate tree species for reforestation of arid and semi-arid regions in tropical and sub-tropical areas. Its potential as an economic crop that may be repeatedly coppiced or harvested provides sustainable unity of ecological and economic benefits. Given the interest in propagating *B. ceiba* and similar crops as a restoration technology for arid and semi-arid regions, we are interested in exploring the mechanisms behind its drought tolerance.

Arbuscular mycorrhizal fungi (AMF) from Glomeromycotina can colonize more than 80% of terrestrial plants (Bonfante, 2018; Wipf et al., 2019). Symbiotic AMF are involved in many functional processes, such as water absorption, nutrient acquisition, carbon metabolism, as well as biotic and abiotic resistance (Wu et al., 2019; Genre et al., 2020; Shi et al., 2021; Zou et al., 2021). AMF has positive effects on the growth and varieties of stress tolerances of host plants (Porcel et al., 2016; Ruiz-Lozano et al., 2016; Zhang et al., 2019; Chu et al., 2021). Thereby mycorrhizal plants can be considered as a potential valuable source against drought stress.

Respiratory burst oxidase homologue (Rboh) proteins, catalyze oxygen to superoxide by oxidizing NADPH. This process is a well-established mechanism for ROS-generation in plants (Torres and Dangl, 2005; Sagi and Fluhr, 2006). Recent studies have implied that Rboh-dependent ROS generation is associated with plant abiotic stress tolerance (e.g., cold tolerance, salt tolerance, metal tolerance, and thermal tolerance etc.) (Miller et al., 2008; Wang et al., 2013; Zhang et al., 2019, 2022), and AMF symbiosis formation (Belmondo et al., 2016; Arthikala et al., 2017). So far, little discussion about *Rboh* response to drought stress associated with AMF.

The detoxification of over-accumulated ROS in plants is maintained by both enzymatic and non-enzymatic antioxidant systems under drought stress (Choudhary et al., 2018). In plant cell, ROS scavenging is done by a variety of enzymatic antioxidants (including superoxide dismutase, (SOD); catalase, (CAT); ascorbate peroxidase, (APX); glutathione reductase, (GR); monodehydroascorbate reductase, (MDHAR); dehydroascorbate reductase, (DHAR); glutathione peroxidase, (GPX); peroxidase, (PX); etc.) and non-enzymatic antioxidants (such as glutathione, (GSH); ascorbic acid, (AsA); etc.) (Hasanuzzaman et al., 2020; Mittler et al., 2022). Largely, drought tolerance is thought of as a measure of ROS detoxification ability through upregulation of one or more of these mechanisms (Fahad et al., 2017; Abideen et al., 2020; Rady et al., 2020). Despite an understanding that AMF-association can increase drought tolerance, the actual mechanisms of this process remain unexplored. Our goal is to explore the differential production and scavenging of ROS in AMF-colonized and non-colonized *B. ceiba* seedlings to further understand its mechanistic metabolic underpinnings. The production and scavenging of ROS are essential factors of plant defense processes, evaluating the antioxidant defense system of AMF-plants and NMF-plants could give an insight into how AMF improved *B. ceiba* seedlings resistance to drought stress.

## Materials and methods

### Plant, substrate and inocula material

Seeds of *Bombax ceiba* L. were collected from a hot-dry valley area (25°40′50.06′′ N, 101°53′27.76′′ E). *Bombax ceiba* seeds were soaked in 30% hydrogen peroxide for 30 min for surface sterilization and then washed 5 times with sterile water. The sterilized seeds were pre-germinated on sterile gauze in Petri dishes (15 cm) at room temperature. During the incubation period, seeds were rinsed with sterilized water twice a day. Germinated seeds were transferred to incubation plates containing autoclaved vermiculite, and grown at 25°C under 14 h day/10 h night conditions. Uniform seedlings (About 6∼7 cm) were selected and individually transplanted into plastic pots (26 cm diameter, 19.5 cm height). Each pot contained an 8 kg homogenized substrate of autoclaved sand, vermiculite, and soil (V/V/V = 1:1:1). The soil was collected from the top layer (10-25 cm) of a nearby forest in Qujing Normal University, Yunnan, China (25°31′38′′ N, 103°44′41′′ E). The sand was washed five times using tap water and dried in the open air before being mixed with soil and vermiculite. After sieving with a 2 mm sieve, the soil and sand were mixed with vermiculite (V/V/V = 1:1:1) and autoclaved at 121°C for 2 h for use as a soil substrate. The physicochemical properties of the soil substrate were pH 7.6 (soil and water ratio were 1:5), the soil available nitrogen, potassium, phosphate and soil organic matter (SOM) are 18.52 mg/kg, 57.63 mg/kg, 9.34 mg/kg and 0.13 g/kg. During seedlings transplanting, 10 mL of mycorrhizal inocula (containing about 226 propagules per milliliter) was added to the root of seedlings. Control seedlings were inoculated with 10 mL of autoclaved inocula and 10 mL of inocula washing solution from live inocula that had been filtered through a 1 μm nylon mesh. The inocula used was the arbuscular mycorrhizal fungus *Rhizophagus irregularis* (Błaszk, Wubet, Renker, and Buscot) Walker & Schüßler (BGC BJ09). This inoculant was obtained from the Beijing Academy of Agriculture and Forestry Sciences, China. The number of propagules per milliliter was determined using the most probable number method (Feldmann and Idczak, 1992).

### Experimental design

The experiment was performed using two water levels (well-watered and drought-stressed) and two AMF treatments (with and without AMF inoculation) as a two-factor experiment, each pot was planted three seedlings, three pots were considered as one replicate unit. Each treatment contained 4 replicates. To experimentally induce drought stress, four weeks after seedlings transplantation, the water content was adjusted to 60% (well-watered) and 20% (drought-stressed) of field capacity and then maintained at corresponding level using TDR 100 monitor daily. All pots were kept at a stable field capacity for 45 days. Throughout the experiment, seedlings were grown in the greenhouse with 35∼24°C temperatures under 14 h daylight and 40∼60% humidity. All pots were irrigated with 50 ml Hoagland solution (Hoagland and Arnon, 1950) every 10 days. Before water stress treatment, all pots were kept with the well-watered treatment.

### Plant sampling and biomass measurement

Plants were harvested after 45 days of the experiment. Shoots and roots of each plant were sampled and the separated shoot and fresh root biomass were weighted. The dry biomass was calculated by the fresh weight and fresh-to-dry mass ratio (Ma et al., 2014). Some root parts were fixed in FAA (37% formaldehyde: glacial acetic acid: 95% ethanol, 9:0.5:0.5, v: v: v) fixative to examine AMF colonization. The rest of the samples were soaked in liquid nitrogen immediately and then stored at −80°C for further analysis.

### Root staining and mycorrhizal colonization quantification

Sample roots were stained with trypan blue (Phillips and Hayman, 1970) following the procedure: 10% KOH for 30 min at 90°C, 10% H_2_O_2_ for 10 min at room temperature, then acidified by 2% HCl for 5 min and stained with trypan blue for 30 min at 90°C. Images were taken using a Leica DM2500 (CMS, GmbH, Wetzlar, Germany) microscope. The mycorrhizal colonization rate of hyphal, arbuscular, vesicle and spore were examined and quantified using the magnified cross sections method (McGonigle et al., 1990).

### Reactive oxygen species and lipid peroxidation measurement

Use potassium phosphate buffer (pH 7.8) to extract roots and shoots powdered samples at 4°C. The production rate of O_2_^.–^ was measured by the method of Ke et al. (2007). The H_2_O_2_ content was measured according to trichloroacetic acid extraction method of Chakrabarty and Datta (2008). H_2_O_2_ standard curve was used to calculate the H_2_O_2_ content. The concentration of malondialdehyde (MDA) was used as determining the level of lipid peroxidation. The content of MDA was measured by the method of Kumar and Knowles (1993).

### Genome-wide identification of *Bombax ceiba* rboh family genes

The whole genome, DNA and protein sequences of *B. ceiba* were downloaded from GigaDB Dataset^1^ (Gao et al., 2018). To identify *Rboh* in *B. ceiba*, the reported ten Rboh protein sequences from *Arabidopsis thaliana* were used as query sequences with an *E*-value cutoff set as 1.0 × e^–10^ to perform a local BLASTP against *B. ceiba* genome. The choice of candidate Rboh was based on the *E*-value, the sequence homology value (>40%) and the value of score (>500). All corresponding cDNA and protein sequences of candidate Rbohs were extracted. All the candidate *BcRboh*s were further confirmed through the Pfam database and the SMART database for conserved domains (including the respiratory burst NADPH oxidase domain, ferric reductases like transmembrane component domain, FAD-binding domain and ferric reductase NAD binding domain) (Sagi and Fluhr, 2006; Oda et al., 2010). Various splicing variants of one gene (which) and the redundant sequences were discarded. The remaining sequences were used in further analyses. The extracted results were subjected to phylogenetic analysis with *Arabidopsis thaliana* and other plant *Rboh* genes. Based on the results of their phylogenetic analysis, the putative *B. ceiba* Rboh named after the *A. thaliana* Rboh name (Table 1).

**TABLE 1:** *Rboh* genes identified in *B. ceiba*.

Gene ID	Gene name	Protein length(aa)	Molecular weight (kDa)	Isoelectric point (PI)	Instability index	GRAVY	Subcellular localization predicted
evm.model.Scaffold412.92	*BcRbohA*	919	104.04	9.19	39.54	−0.311	plas: 5, nucl: 3, chlo: 2, cyto: 2, vacu: 1
evm.model.Scaffold167.494	*BcRbohB*	886	100.73	9.28	41.78	−0.295	plas: 5, cyto: 4, nucl: 3, chlo: 1
evm.model.Scaffold63.66	*BcRbohC*	927	105.04	9.20	40.27	−0.265	plas: 5, chlo: 2, cyto: 2, nucl: 1, mito: 1, E.R.: 1, pero: 1
evm.model.Scaffold92.69	*BcRbohD*	929	104.64	9.07	37.44	−0.29	8, E.R.: 4, nucl: 1
evm.model.Scaffold230.154	*BcRbohDX1*	940	105.81	9.00	40.50	−0.273	plas: 8, E.R.: 5
evm.model.Scaffold4.238	*BcRbohDX2*	933	105.08	9.31	40.45	−0.229	plas: 8, chlo: 2, cyto: 2, nucl: 1
evm.model.Scaffold74.255	*BcRbohE*	936	106.59	9.03	50.87	−0.251	plas: 8, E.R.: 3, nucl: 1, mito: 1
evm.model.Scaffold220.422	*BcRbohF*	932	106.32	9.39	44.64	−0.249	cyto: 12, nucl: 1
evm.model.Scaffold64.375	*BcRbohFX1*	927	105.48	9.37	47.66	−0.215	cyto: 6, chlo: 4, nucl: 3
evm.model.Scaffold168.218	*BcRbohG*	917	103.48	8.99	38.35	−0.28	nucl: 5, E.R.: 3, plas: 2, chlo: 1, cyto: 1, vacu: 1
evm.model.Scaffold25.171	*BcRbohH*	905	103.16	9.31	46.26	−0.209	plas: 5, nucl: 4, cyto: 4
evm.model.Scaffold145.120	*BcRbohHX1*	890	101.93	9.22	46	−0.214	plas: 10, nucl: 3
evm.model.Scaffold89.322	*BcRbohI*	930	106.03	9.35	48.99	−0.205	cyto: 11, nucl: 3
evm.model.Scaffold63.252	*BcRbohJ*	802	91.58	9.22	49.19	−0.133	plas: 7, E.R.: 3, nucl: 2, mito: 1

Plas indicate plasma membrane, E. R. indicate endoplasmic reticulum membrane, cyto indicate cytoplasmic, nucl indicate nuclear, mito indicate mitochondrial, pero indicate glyoxysomal, golg indicate golgi apparatus, vacu indicate vacuoles, and chlo indicate chloroplast.

### Rboh protein properties, subcellular location and multiple sequence alignment and phylogenetic analysis

Protein properties such as hydropathicity, isoelectric point, instability index and molecular weight were predicted using the ExPASy-ProtParam tool^2^. The subcellular localizations of *BcRboh* proteins were analyzed using WoLF PSORT^3^. Multiple amino acids of *BcRboh*s were aligned and colored in DNAMAN7.0^4^ (Supplementary Figure 1). The phylogenetic tree was constructed using the maximum likelihood method employing ClustalW with default parameters, JTT matrix-based model, pairwise gap deletion with the 1,000 bootstrap in MEGA 7^5^.

### Gene expression profile of BcRbohs in *Bombax ceiba*

Total RNA was extracted from shoots and roots using a Plant RNA extraction kit (Omega Bio-Tek, Norcross, GA, USA) according to the manufacturer’s instructions. RNA concentrations were measured by NanoDrop 2000 (Thermo Scientific, Pittsburgh, PA, USA). RNA quality was verified by performing agarose gel electrophoresis. First-strand cDNA synthesis was obtained by using a PrimerScript^®^ RT Reagent Kit with gDNA Eraser (TaKaRa Bio, Dalian, China) following the supplier’s protocol.

To obtain expression profiles for *BcRboh*s in root and shoot in response to drought-stressed treatment, we performed quantitative real-time PCR (qRT-PCR) on a Roche LightCycle 96 machine (Roche, Germany), using SYBR Green qPCR kits (TaKaRa) according to the manufacturer’s instructions. The Actin gene served as our standard. Gene-specific primers were designed for these amplifications (Supplementary Table 1). All reactions included 10.0 μL of SYBR R Premix Ex Taq TM (TaKaRa), 5.0 μL of tenfold diluted cDNA as template, 0.5 μL of each specific primer, and 4 μL of ddH_2_O, made up to a 20 μL volume. qPCR was performed under the following thermal cycles: initial 95°C for 3 min; then 40 cycles of 95°C for 20 s, 56°C for 20 s, and 72°C for 20 s. Based on four separate RNA extracts from four biological replications samples, each qRT-PCR was conducted three times to minimize inherent errors. The relative expression levels of all *BcRboh* genes were calculated by the 2^–ΔΔCT^ method (Livak and Schmittgen, 2001).

### Antioxidant system activities measurement

Powdered samples were completely homogenized with an extraction buffer (contained 1% polyvinylpyrrolidone (PVP), 1 mM EDTA and 50 mM potassium phosphate buffer), and the homogenized liquids were centrifuged at 14,000 g for 30 min at 4°C. The supernatant was taken and used to determine the antioxidant enzyme activity (SOD, PX, CAT, APX, GPX, GR, MDAR, and DHAR) and total soluble protein in each sample. SOD activity was determined using inhibition of the photochemical reduction of nitroblue tetrazolium (Beyer and Fridovich, 1987). The PX activity was determined using a modified method of Amako et al. (1994) and measuring the catalyzed oxidation of guaiacol to tetraguaiacol. A five-mute H_2_O_2_ decomposition, was used to measure CAT activity through the proxy of the absorbance of OD_240_. Decomposition was carried out at pH 7.0 and 25°C (Aebi, 1984). The ascorbate oxidation rate, measured by the absorbance of OD_290_, during a pH 7.0 and 20°C 3 min H_2_O_2_ decomposition, was used as a proxy for APX activity (Nakano and Asada, 1981). The GR activity was determined by the NADPH oxidation rate according to the reduction absorbance of OD_340_ for 3 min (Meloni et al., 2003). The GPX activity was determined by the guaiacol oxidation rate (Flohé and Günzler, 1984). The MDAR activity was determined by the reduction absorbance of OD_340_ nm for 3 min (Hossain et al., 1984). The DHAR activity was measured by the method of Kubo et al. (1999). Total soluble protein was determined with Xylene brilliant cyaninG as the color agent, and bovine serum albumin was used as a standard, following the method of Sedmak and Grossberg (1977).

Powdered samples were homogenized with 5% trichloroacetic acid at 4°C and centrifuged at 16,000 g for 15 min to produce a supernatant. The supernatant was prepared to measure the reduced glutathione (GSH) and glutathione disulfide (GSSG) concentrations. The reduction in the sulfhydryl reagent 5,5′-dithio-bis (2- nitrobenzoic acid) (DTNB) measured at 412 nm was used as the GSH concentration (Rahman et al., 2006). GSSG can be reduced to GSH by GR in the presence of NADPH. The concentration of GSSG was calculated by the sum of the content of GSH and the content of reduced GSSG minus the GSH content.

The ascorbic acid (AsA) and dehydroascorbic acid (DHA) were measured according to the methods described by Zhang et al., 2019. Powdered samples were also homogenized with 5% metaphosphoric acid and centrifuged at 16,000 g for 15 min in 4°Cto determine the AsA and DHA concentrations. The reduction of Fe^3+^ measured by the absorbance of OD_525_ was used as AsA concentration (Zhang and Kirkham, 1996). DHA can be reduced to AsA by the reduction of dithiothreitol, and the total AsA that included DHA and AsA was measured. The concentration of DHA was calculated by subtracting AsA concentration by the total AsA concentration.

### Statistical analysis

Statistical analysis was performed using the IBM SPSS 19.0 statistical program (SPSS Inc., Chicago, IL, USA). The data from the experiment were analyzed using a two-way ANOVA with two factors (AMF inoculation and water stress) followed by Duncan’s test when the ANOVA was significant. The cluster analysis of ROS and antioxidant system parameters and the relative expression of *BcRboh*s genes were analyzed by TBtools v1.098726 (Chen C. et al., 2020). The PCA of the antioxidant system parameters was analyzed using factor analysis after the sphericity test of KMO and Bartlett in SPSS.

## Results

### Biomass and colonization

**FIGURE 1:**
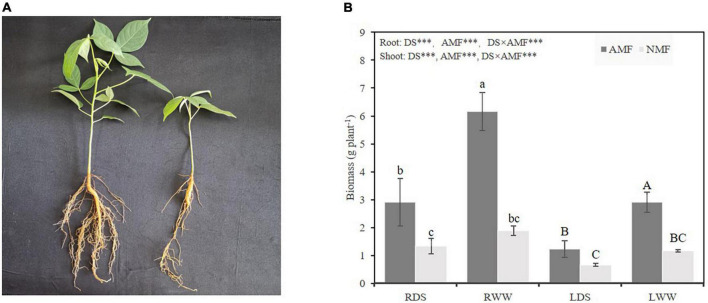
**(A)** Photo of *Bambax ceiba* inoculated with AMF (left), and non-inoculated with AMF (right). **(B)** The dry weight both in shoots and roots of *B. ceiba* plants inoculated with/without the AMF *R. irregularis* under drought stress. The data are the means ± standard deviation (*n* = 4). Different small and capital letters above the columns indicate significant difference among the means by Duncan’s test (*P* < 0.05), respectively. Significant effect of two-way ANOVA ***P < 0.001, NS, no significant effect. NMF indicate non-mycorrhizal treatment; AMF indicate arbuscular mycorrhizal fungi inoculation; DS indicate drought-stressed treatment; RDS indicate root of drought-stressed treatment; RWW indicate root of well-watered treatment; LDS indicate shoot of drought-stressed treatment; LWW indicate shoot of well-watered treatment.

**FIGURE 2:**
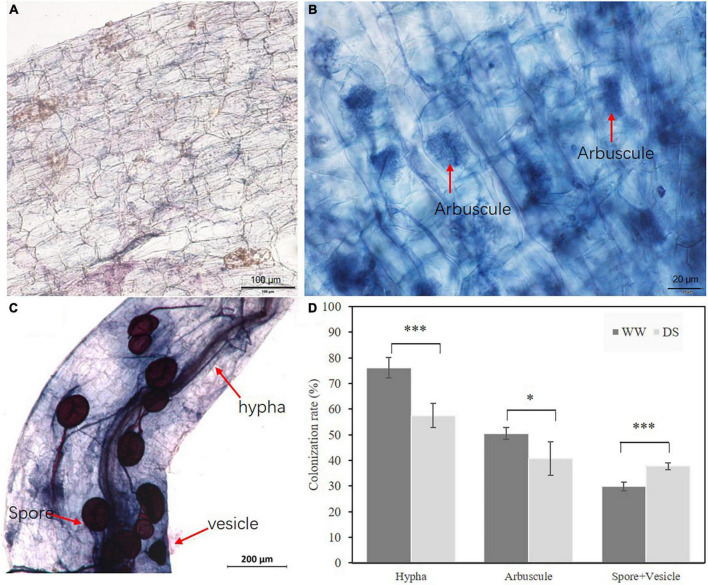
Root colonization characters of AMF *R. irregularis.* **(A)** *B. ceiba* root of NMF treatment. **(B)** Arbuscule of AMF *R. irregularis* in *B. ceiba* root. **(C)** Spore of AMF *R. irregularis* in *B. ceiba* root. **(D)** The colonization rate of *R. irregularis* in mycorrhizal plants under drought stress. The data are the means ± standard deviation (*n* = 4). Significant difference between DS and WW was tested by Student’s T test. *P < 0.05, ***P < 0.001, DS indicate drought-stressed treatment, WW indicate well-watered treatment. Hypha indicate hypha colonization rate; Spore+Vesicle indicate spore and vesicle and colonization rate; Arbuscule indicate arbuscule colonization rate.

AMF plants grew better than NMF plants at both drought-stressed and well-watered treatments (Figure 1A). In the drought-stressed treatment, AMF inoculation increased root and shoot weights by 118.80, 86.84%, respectively. AMF inoculation also increased root and shoot weights by 227.66, and 150.69%, respectively, in ‘unstressed’ well-watered plants. As expected, drought stress decreased the roots and shoots dry weight by 41.35 and 75.79% compared with well-watered treated plants in NMF treatment, respectively. Similarly, drought stress decreased the roots and shoots dry weight by 111.68 and 135.87% in AMF treatment (Figure 1B). To further confirm the difference in biomass was caused by AMF inoculation, AMF colonization rate was checked. No mycorrhizal colonization was observed in NMF plants in either the well-watered or drought-stressed treatment (Figure 2A). Typical mycorrhizal structures like arbuscules, vesicles and inter-radical spores were found in AMF plants in both drought-stressed and well-watered treatments (Figures 2B,C). Compared with well-watered AMF plants, drought stress significantly decreased hypha and arbuscule colonization but increased spore and vesicle colonization (Figure 2D).

### Reactive oxygen species levels and oxidative damage to lipids

**FIGURE 3:**
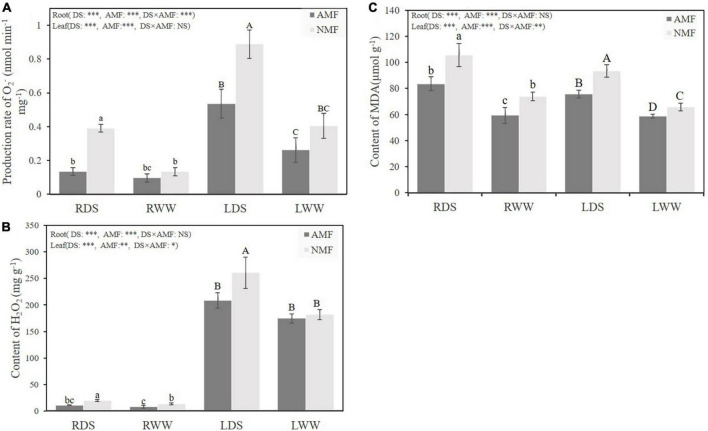
**(A)** The O_2_⋅^–^ generation rate in roots and shoot of *B. ceiba* plants inoculated with/without the AMF *R. irregularis* under well-watered and drought-stressed treatments; The abbreviation is consistent with the above Figure 1 **(B)**. **(B)** The H_2_O_2_ content in roots of *B. ceiba* plants inoculated with/without the AMF *R. irregularis* under well-watered and drought-stressed treatments. **(C)** The levels of MDA content in roots and shoot of *B. ceiba* plants inoculated with/without the AMF *R. irregularis* under well-watered and drought-stressed treatments. The data are the means ± standard deviation (*n* = 4). Different small and capital letters above the columns indicate significant differences among the means by Duncan’s test (*P* < 0.05). Significant effect of two-way ANOVA: *P < 0.05, **P < 0.01, ***P < 0.001, NS, no significant effect.

Drought stress significantly increased the O_2_⋅^–^ generation rate of both NMF plants and AMF plants in root and leaf tissues, respectively. Interestingly, AMF-inoculated plants presented lower rates of O_2_⋅^–^ generation rate of root and shoot under drought stress by 65.20, 39.69%, for root and shoot tissue, respectively (Figure 3A). Drought stress also increased H_2_O_2_ content by 44.51 and 43.40% in root and leaf of non-inoculated NMF plants. Conversely, AMF-inoculated plants did not show increased H_2_O_2_ generation under the drought treatment. AMF inoculated plants had 43.21% and 20.07% lower in root and leaf H_2_O_2_ content levels than their non-colonized counterparts. Intriguingly, H_2_O_2_ content in leaf was 13 to 22-fold compared with the H_2_O_2_ content in root of AMF or NMF under both well water and drought stress treatments (Figure 3B). In this study, drought stress substantially increased the MDA content of both AMF and NMF plants. Among all plants, however, MDA content was lower in AMF-inoculated individuals, regardless of water treatment. AMF-inoculated plants had lower levels of the ROS H_2_O_2_ under well-watered condition as well. Well-watered AMF-inoculated plants had 19.72 and 10.74% less H_2_O_2_ than non-mycorrhizal counterparts in root and leaf tissues, respectively (Figure 3C). Totally, these results indicate that inoculation of AMF decreased the O_2_⋅^–^ generation rate, the H_2_O_2_ content and the MDA content under drought stress.

### Identification and phylogenetic analysis of the BcRboh genes

Totally, 14 candidate *BcRboh* genes were identified from the *B. ceiba* genome by local BLASTP searches. Protein domain identification was performed by Simple Modular Architecture Research Tool (SMART) (Letunic et al., 2021). Four conserved domains were found, including NADPH-Ox and Ferric-reduct in the *N*-terminal region and FAD-binding-8 and NAD-binding-6 in the C-terminal region (Supplementary Figure 1). The identity of nucleotide and amino acid ranges from 52.00 to 92.00 and 42.50 to 92.90%, respectively (Supplementary Table 2). The putative BcRboh proteins consisted of 802-940 amino acids, with calculated molecular weights from 91.58 to 106.59 kDa, and their isoelectric points (PI) ranged from 8.99 to 9.39. The Grand average of hydropathicity (GRAVY) values of all putative BcRboh were negative and ranged from −0.311 to −0.133, indicating that all these Rboh proteins were hydrophilic (Gasteiger et al., 2005). Their instability index showed that except for BcRbohA, BcRbohD, and BcRbohG, the others were instability (Table 1).

**FIGURE 4:**
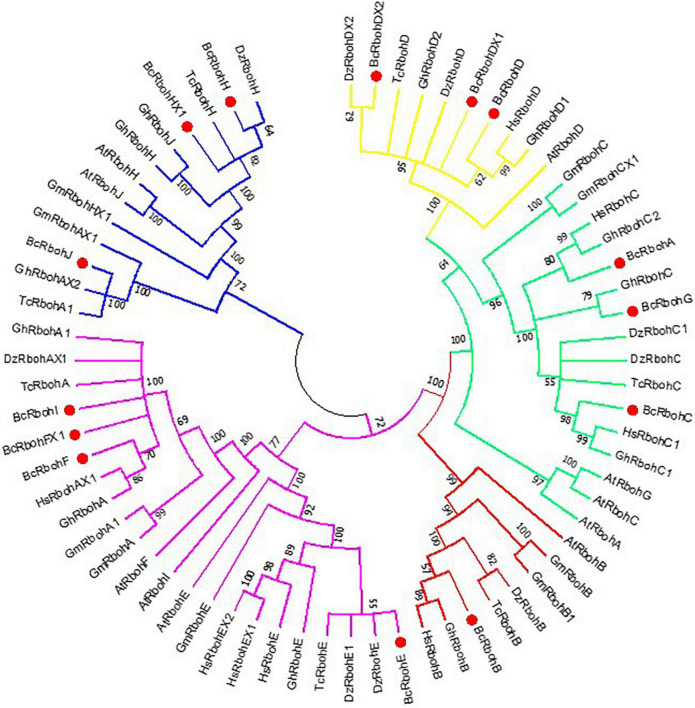
Phylogenetic analysis of *B. ceiba* Rboh and Rboh homologs of other plant. An unrooted circle phylogenetic tree of the plant Rboh proteins was constructed using the maximum likelihood method with MEGA 7.0 program. Numbers next to branches represent the percentages of replicate trees in which the associated taxa clustered together in the bootstrap test (10,000 replicates). Sequence names consist of species code (first letter of genus, second letter of species name) and gene name (At abbreviation of *Arabidopsis thaliana*, Gm abbreviation of *Glycine max*, Dz abbreviation of *Durio zibethinus*, *Tc* abbreviation of *Theobroma cacao*, Gh abbreviation of *Gossypium hirsutum* and *Hs* abbreviation of *Hibiscus syriacus*). Accession numbers of the predicted proteins are given in Supplementary materials (Supplementary Table 3). Group 1 subtree of yellow line, Group 2 subtree of green line, Group 3 subtree of red line, Group 4 subtree of purple line, Group 5 subtree of blue line.

To understand the evolutionary relationship among the Rbohs in *B. ceiba*, putative BcRboh protein sequences and Rboh protein sequences of other plants (*A. thaliana*, *Glycine max*, *Durio zibethinus*, *Theobroma cacao*, *Gossypium hirsutum*, and *Hibiscus syriacus*) were used to construct a phylogenetic tree (Figure 4). According to the phylogenetic tree, BcRbohs were clustered into five groups, and the BcRbohs were distributed in each group. The BcRbohD, BcRbohDX1, and BcRbohDX2 belonged to Group 1; BcRbohA, BcRbohC, and BcRbohG were classified into Group 2; BcRbohB were part of Group 3; BcRbohE, BcRbohF, BcRbohFX1, and BcRbohI were categorized into Group 4; BcRbohH, BcRbohHX1, and BcRbohJ were assigned into Group 5 (Figure 4).

### Responses of BcRboh expression to arbuscular mycorrhizal fungi inoculation

**FIGURE 5:**
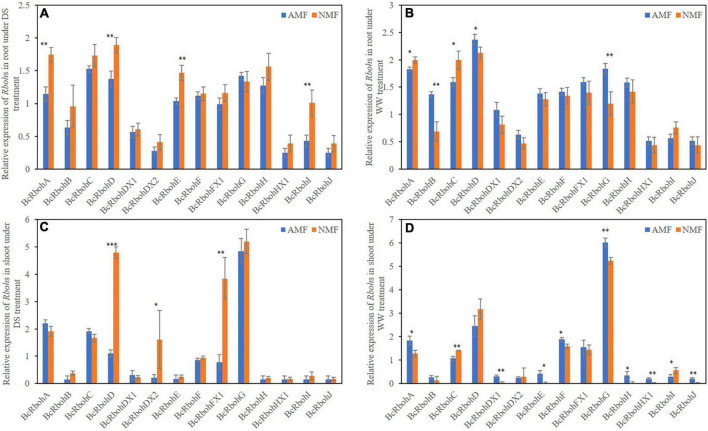
**(A)** Relative expression of Rbohs in root under DS treatment. **(B)** Relative expression of Rbohs in root under WW treatment. **(C)** Relative expression of Rbohs in shoot under DS treatment. **(D)** Relative expression of Rbohs in shoot under WW treatment. Relative expression of 14 *BcRboh* genes in root and shoot of *B. ceiba* seedlings that received the AMF and drought stress treatments. The relative expression of *BcRbohHX1* received well-watered with NMF treatment in shoot as a standard (0). The data are the means ± standard deviation (*n* = 4). Significant difference between AMF and NMF was tested by *Student’s T* test. *P < 0.05, **P < 0.01, ***P < 0.001. No star symbol above the column indicate no significant. DS indicate drought-stressed treatment, WW indicate well-watered treatment.

The effect of AMF on mRNA levels of *BcRboh* were measured. Under drought stress, *BcRbohA, BcRbohD, BcRbohE, BcRbohI* were downregulated and no genes were upregulated in root by AMF colonization (Figure 5A). AMF-inoculated plants upregulated the expression of *BcRbohB*, *BcRbohD*, *BcRbohG* and downregulated the expression of *BcRbohA*, *BcRbohC* expression in root tissues of well-watered plants (Figure 5B). In shoot, the relative expression of *BcRbohD*, *BcRbohDX2* and *BcRbohFX1* were downregulated and no genes were upregulated by AMF inoculation under drought stress (Figure 5C). Except *RbohC* and *RbohI*, the expression of most *Rboh* genes (*RbohA*, *RbohDX1*, *RbohE*, *RbohF*, *RbohG*, *RbohH*, *RbohHX1*, and *RbohJ*) were slightly upregulated in well-watered AMF-inoculated plants compared to their non-inoculated neighbors (Figure 5D). The relative expression level showed difference among these *BcRboh* genes. Thus, there was an extensive variation in *BcRboh* expression levels in response to AMF inoculation and drought stress (Figure 5).

### Response of antioxidant systems parameters to DS and arbuscular mycorrhizal fungi inoculation

Drought stress increased SOD, PX, APX, GPX, GR, MDAR, DHA, and GSSG by 20.87, 24.69, 161.17 16.48, 70.80, 160.77, 31.04 and 123.12% in root tissues of NMF plants. In NMF leaf tissues, drought stress increased SOD, CAT, APX, GPX, GR, MDAR, and GSSG by 84.12%, 170.41, 64.04, 18.74, 28.65, 28.61 and 38.66%, but decreased AsA, AsA/DHA. Similarly, drought stress increased SOD, PX, CAT, APX, GPX, GR, MDAR, DHAR by 101.81 and 123.72%, 101.53 and 8.82%, 90.31 and 318.53%, 63.75 and 34.11%, 175.25 and 16.29%, 29.53 and 22.75, 219.29 and 82.20%, 136.88 and 73.70% in both root and leaf in AMF plants. Under drought stress, AMF inoculation increased SOD, PX, CAT, APX, GPX, MDAR, DHAR by 87.09 and 22.95, 41.40, and 25.19%, 26.34 and 21.54%, 15.03 and 19.47, 66.80, and 12.83%, 80.49 and 19.50%, 131.50 and 92.33% in root and shoot, respectively. GR, AsA, GSH, AsA/DHA, GSH/GSSG in root were also increased by AMF inoculation by 49.61, 28.16, 154.87, 71.21, 462.61% under drought stress. Furthermore, under well-watered conditions, APX, GSH in root and leaf, GPX, MDAR, AsA, GSSG, AsA/DHA in root and DHA, GSH/GSSG in shoot were higher in AMF-inoculated plants than their non-inoculated counterparts (Table 2).

**TABLE 2:** The activities of antioxidant defense of mycorrhizal and non-mycorrhizal roots and shoots under drought stress.

Treatment	Loc	NMF + DS	AMF + DS	NMF + WW	AMF + WW	*P*_*AMF*_	*P*_*DS*_	*P_*AMF*×*DS*_*
SOD (U min^–1^ mg^–1^ protein)	Root	17.76 ± 0.72b	33.23 ± 2.33a	14.69 ± 0.55c	16.46 ± 0.57bc	***	***	***
	Leaf	22.64 ± 2.95B	27.84 ± 1.45A	12.29 ± 2.06C	12.44 ± 1.04C	**	***	**
PX (U min^–1^ mg^–1^ protein)	Root	17.84 ± 0.91b	25.23 ± 2.39a	14.31 ± 0.63c	12.52 ± 1.64c	**	***	***
	Leaf	2.70 ± 0.34AB	3.38 ± 0.47A	2.36 ± 0.51B	3.10 ± 0.56AB	*	NS	NS
CAT (U mg^–1^ protein)	Root	2.05 ± 0.31b	2.59 ± 0.26a	1.99 ± 0.35bc	1.36 ± 0.29c	NS	***	**
	Leaf	17.92 ± 1.38B	21.78 ± 1.65A	6.63 ± 0.82C	5.20 ± 0.74C	NS	***	**
APX (U mg^–1^ protein)	Root	19.63 ± 1.95a	22.58 ± 2.45a	7.52 ± 0.96c	13.79 ± 1.53b	***	***	NS
	Leaf	15.41 ± 1.63B	18.41 ± 1.78A	9.37 ± 1.02C	13.73 ± 1.68B	**	***	NS
GPX (U min^–1^mg^–1^ protein)	Root	50.15 ± 2.78b	83.65 ± 6.13a	43.06 ± 2.82c	30.39 ± 2.58d	***	***	***
	Leaf	42.67 ± 2.09B	48.14 ± 2.31A	35.93 ± 2.08C	41.40 ± 3.16B	**	***	NS
GR (U mg^–1^ protein)	Root	19.39 ± 1.38b	29.01 ± 1.69a	16.98 ± 0.89c	14.97 ± 0.92d	***	***	***
	Leaf	21.33 ± 1.05A	22.53 ± 0.90A	16.58 ± 1.28B	18.36 ± 1.57B	*	***	NS
MDAR (U mg^–1^ protein)	Root	3.49 ± 0.59b	6.30 ± 0.44a	1.33 ± 0.09d	1.97 ± 0.35c	***	***	***
	Leaf	7.20 ± 0.86B	8.61 ± 0.73A	5.20 ± 0.47C	4.72 ± 0.24C	NS	***	*
DHAR (U mg^–1^ protein)	Root	12.81 ± 1.27b	29.66 ± 2.88a	11.22 ± 0.72b	12.52 ± 0.84b	***	***	***
	Leaf	1.99 ± 0.74B	3.82 ± 0.75A	1.55 ± 0.22B	2.20 ± 0.29B	**	**	NS
AsA (μg g^–1^ FW)	Root	24.31 ± 1.24c	31.16 ± 4.44b	34.98 ± 2.30b	47.90 ± 6.18a	***	***	NS
	Leaf	62.58 ± 8.82C	73.98 ± 11.16BC	90.71 ± 7.63A	78.22 ± 6.60AB	NS	**	*
DHA (μg g^–1^ FW)	Root	94.97 ± 6.03a	70.57 ± 7.41c	72.47 ± 4.00bc	83.88 ± 11.85ab	NS	NS	**
	Leaf	125.86 ± 9.61B	171.20 ± 17.34A	138.94 ± 17.21B	169.77 ± 15.58A	***	NS	NS
GSH (μg g^–1^ FW)	Root	0.71 ± 0.05b	1.80 ± 0.26a	0.69 ± 0.08b	1.77 ± 0.31a	***	NS	NS
	Leaf	1.41 ± 0.14B	1.76 ± 0.22AB	1.50 ± 0.24B	2.12 ± 0.37A	NS	NS	**
GSSG (μg g^–1^ FW)	Root	1.51 ± 0.07a	0.71 ± 0.16b	0.68 ± 0.10b	1.41 ± 0.13a	NS	NS	***
	Leaf	1.70 ± 0.24A	1.43 ± 0.09AB	1.32 ± 0.24B	0.89 ± 0.11C	**	***	NS
AsA/DHA	Root	0.26 ± 0.03c	0.44 ± 0.02b	0.48 ± 0.09b	0.57 ± 0.04a	***	***	**
	Leaf	0.50 ± 0.05B	0.43 ± 0.04B	0.66 ± 0.13A	0.46 ± 0.03B	**	*	NS
GSH/GSSG	Root	0.47 ± 0.04c	2.64 ± 0.75a	1.03 ± 0.13bc	1.25 ± 0.11b	***	NS	***
	Leaf	1.23 ± 0.22B	0.85 ± 0.78B	1.16 ± 0.26B	2.44 ± 0.66A	**	**	**

The data are the means ± standard deviation (*n* = 4). Different small and capital letters indicate significant differences among the means by Duncan’s test (*P* < 0.05), Significant effect of two-way ANOVA: *0.01 < *P* < 0.05, **0.001 < *P* < 0.01, ***P < 0.001, NS, no significant effect. Loc indicate location, NMF + DS indicate non-inoculated AMF in drought stress treatment, NMF + WW indicate non-inoculated AMF in well-watered treatment, AMF + DS indicate AMF inoculation in drought stress treatment, AMF + WW indicate AMF inoculation in well-watered treatment.

### Principal component analysis and cluster analysis of reactive oxygen species and antioxidant-related parameters

**FIGURE 6:**
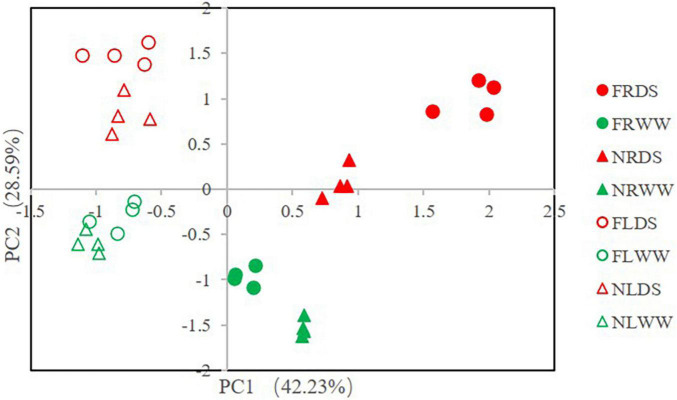
Principle component analysis (PCA) of reactive oxygen species (ROS) and antioxidant systems parameters. FR, root of inoculation of AMF treatment; NR, root of NMF treatment; FL, shoot of inoculated AMF treatment. DS indicate drought-stressed treatment, WW indicate well-watered treatment.

**FIGURE 7:**
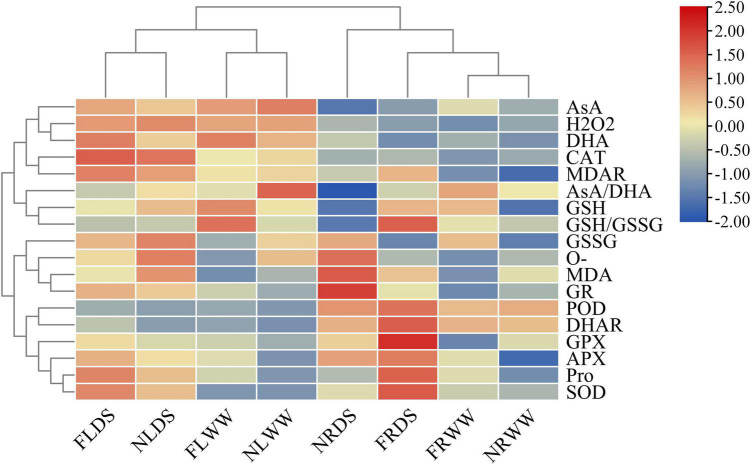
Heatmap of ROS and antioxidant systems parameters. FR, root inoculated with AMF; NR, root inoculated with inactive AMF; FL, shoot of inoculated AMF treatment; NL, shoot of inoculated inactive AMF treatment. DS indicate drought-stressed treatment, WW indicate well-watered treatment. SOD, Superoxide dismutase; PX, peroxidase; CAT, catalase; APX, ascorbate peroxidase; GPX, glutathione peroxidase; GR, glutathione reductase; MDHAR, monodehydroascorbate reductase; DHAR, dehydroascorbate reductase; AsA, ascorbic acid; DHA, dehydroascorbic acid; GSH, glutathione; and GSSG, glutathione disulfide.

The principal component analysis (PCA) of ROS and antioxidant-related parameters revealed the effects of drought stress and AMF (Figure 6). PC1 accounted for 42.23% of the variance and that PC2 accounted for 28.59% of the variance. Root samples inoculated with AMF under drought stress were most different from leaf samples without AMF under well water. Root and leaf were separated by PC1, drought-stressed and well-watered treatments samples could separate from each other. AMF-treatment samples also separated from NMF-treatment samples in root. Cluster analysis of the ROS and antioxidant-related parameters revealed that all samples clustered into two groups (root and shoot groups). And the leaf clade clustered into two subclades by samples of DS and WW (Figure 7). In root clade, AMF and NMF samples under well-watered treatment were clustered together and separated from drought stress, which indicates AMF played a more important role under drought stress.

## Discussion

Drought stress is a vital abiotic stress to plant and worldwide distribution (Piao et al., 2010; Trenberth et al., 2014). AMF have been considered to play a vital role in plant resistance to a variety of abiotic and biotic stresses including drought stress (Wu et al., 2013; Mitra et al., 2021; Usman et al., 2021; Zou et al., 2021). In our study, AMF-colonized *B. ceiba* grew better than non-mycorrhizal plants under both drought-stressed or well-watered treatments. This result suggests that AMF inoculation increases drought stress tolerance of *B. ceiba*.

Drought stress provokes plant photosynthetic dysfunction by stomatal closure, impaired gas exchange, imbalance in the light harvest and utilization, and altered photochemistry in chloroplasts, leading to ROS overproduction (Müller et al., 2001; Augé et al., 2015; Li J. et al., 2019; Hasanuzzaman et al., 2021). Moreover, denaturation of membrane and protein from photorespiration, inactivation of TCA cycle enzymes, and reduced carboxylation efficiency during drought stress can also be linked to ROS overproduction (Cruz de Carvalho, 2008; Hasanuzzaman et al., 2013; Mittler et al., 2022). Additionally, lower NADP^+^ regeneration causes a greater reduction of ETC under drought stress leading to higher electrolyte leakage, ultimately resulting in excess ROS metabolism and oxidative stress (Fahad et al., 2017; Hasanuzzaman et al., 2018, 2020). Thus, drought stress leads to a disbalance of ROS metabolism. As one of the main productions of ROS, the generation rate of O_2_⋅^–^ was significantly increased by drought stress in root and shoot under both AMF and NMF treatments of *B. ceiba* seedlings, which was expected (Chen W. et al., 2020). AMF inoculation, however, correlated with a decreased O_2_⋅^–^ generation rate in root and shoot of *B. ceiba* seedlings under drought conditions (Figure 3A). This result indicates that AMF inoculation could relieve the ROS damage by decreasing the O_2_⋅^–^ generation rate caused by drought stress. Mycorrhizal-colonized-plants also had lower relative electrolyte leakage (Arthikala et al., 2015; Huang et al., 2020), which could contribute to a lower O_2_⋅^–^ production rate and explain the variation between AMF-associated and non-colonized plants. Chen W. et al. (2020) also found that AMF alleviated drought-induced oxidative stress by attenuating the excess generation of O_2_⋅^–^ in the leaves of *Catalpa bungei*.

AMF-colonization may result in better ROS regulation in both stressed and unstressed plants, allowing for AMF-colonized plants to seize a fitness advantage when stressed by ROS-metabolism destabilizing drought conditions. For example: ROS homeostasis is regularly evaluated by MAD content (Zhang et al., 2019). Mycorrhizal seedlings have a lower accumulated MDA content in both root and shoot under well-watered and drought-stressed treatments than non-mycorrhizal seedlings. This matches with the established literature. For example, Huang et al. (2020) found that mycorrhizal plants in general had lower accumulated MDA, H_2_O_2_, and O_2_⋅^–^ than non-mycorrhizal apple seedlings. Therefore, non-mycorrhizal plants suffered higher oxidative damage than AMF plants. Among all ROS, H_2_O_2_ commonly acts as one of versatile molecule that acts as a signal at a normal level, but induces oxidative stress at an abnormal level under drought stress or other stress (Hasanuzzaman et al., 2020; Mittler et al., 2022). In our study, AMF inoculation decreased the content of H_2_O_2_ in roots and shoots under drought stress. In well-watered treatments, AMF only decreased H_2_O_2_ content in root, but does not affect shoots. However, studies showed that H_2_O_2_ generation in mycorrhiza-containing cortical cells ensures the initial AMF colonization in roots, and accumulates in the arbuscule-containing root cortical cells (Salzer et al., 1999; Kapoor and Singh, 2017). This might be because the mycorrhizal-colonized seedlings had relatively higher net root H_2_O_2_ effluxes than non-mycorrhizal seedlings (Huang et al., 2017) which lead to a lower H_2_O_2_ content in mycorrhizal-seedlings, potentially protecting against drought stress. Lanfranco et al. (2005) found that CuZn-SOD was up-regulated in arbuscule-containing cells, which might act as a protection mechanism to decrease the H_2_O_2_ induced in plants. In addition, H_2_O_2_ content in leaf was 13 to 22-fold compared the H_2_O_2_ content in root of AMF or NMF under both well-watered and droughts-stressed treatments. This might because of ROS overproduction caused by photosynthetic dysfunction, imbalance in the light harvest and utilization in chloroplasts under drought stress are located in leaves (Mittler et al., 2022). Furthermore, the main root of *B. ceiba* seedlings are swelling (Figure 1A), which diluted the high H_2_O_2_ content in cortical cells of root. In total, AMF inoculation dramatically decreased H_2_O_2_, O_2_⋅^–^, and MDA concentrations in shoots and roots in *B. ceiba* seedlings under drought stress, relieved the ROS damage caused by drought stress. Huang et al. (2017) also found that the H_2_O_2_, O_2_⋅^–^, and MDA concentrations in leaves and roots were dramatically lower in mycorrhizal trifoliate orange seedlings than in non-mycorrhizal seedlings under drought stress.

*Rboh*s are responsible for ROS generation and are involved in regulating a diverse range of biological processes of various biotic and abiotic stresses including drought stress responses in plants (Sagi and Fluhr, 2006, Chapman et al., 2019, Tarawneh et al., 2020; Zhang et al., 2022). In this study, fourteen *BcRboh* genes members were identified in the *B. ceiba* genome. Prediction programs for subcellular localization showed that the fourteen BcRboh proteins were localized in the plasma membrane, endoplasmic reticulum membrane, etc. indicating different cellular functions (Li D. et al., 2019; Huang et al., 2021). Analysis of the domain composition of BcRbohs revealed that members of the *BcRboh* genes family were relatively conserved during evolution (Supplementary Figure 1), which is consistent with other studies (Sagi and Fluhr, 2006; Zhang et al., 2021; Zhang et al., 2022). Phylogenetic analysis demonstrated that *BcRboh*s were clustered into five groups, consistent with earlier reports (Zhang et al., 2021, 2022). Sequences clustered in the same phylogenetic subclade usually had a close evolutionary relationship, conserved gene structures and similar functions. Genome-wide association mapping reveals that the genes for the plant *Rboh* family might be involved in the tolerance of drought stress (Tarawneh et al., 2020). Among the ten *AtRboh* genes, *AtRbohD* shows a high degree of stress responsiveness both in shoots and roots (Suzuki et al., 2011). In our study, *BcRbohD*, *BcRbohDX1* and *BcRbohD*X2 were down-regulated by drought stress in root of AMF treatment. Besides, NADPH oxidase (RBOH) is the main pathway for H_2_O_2_ production in plants (Hasanuzzaman et al., 2020). The transcriptional levels of *BcRbohA*, *BcRbohC* were lower in AMF treated plant than non-AMF-inoculated plants in well-watered conditions. Under drought conditions, AMF-inoculated plants had decreased *BcRbohA*, *BcRbohD*, *BcRbohE* and *BcRbohI* expression in root tissues than non-mycorrhizal plants. Correspondingly, AMF-inoculated plants also had reduced levels of H_2_O_2_ than their non-mycorrhizal partners. Consistent with the transcriptional levels of *RbohD*, *RbohDX2*, and *RbohFX1*, the content of H_2_O_2_ was lower in AMF shoots than NMF shoots under drought stress treatment. In another hand, studies showed that the expression of *Rboh* in plants play an important role in AMF symbiosis (Belmondo et al., 2016; Zhang et al., 2019; Zhou et al., 2019). Arthikala et al. (2014) found that overexpression *RbohB* of *Phaseolus vulgaris* impaired AMF colonization. Zhang et al. (2019) found that inhibition of NADPH oxidase activity (DPI treatment) stimulated arbuscule colonization. However, *MtRbohE* was activated in arbuscular cells involved in root cortex colonization (Belmondo et al., 2016). Arthikala et al. (2013) suggested that NADPH oxidase induces H_2_O_2_ burst in AMF roots, is was related to the interaction between AMF and host plants. In our study, AMF decreased transcriptional levels of *BcRboh* genes under drought stress thereby lower H_2_O_2_ content in AMF plant. These results suggest that RBOH-mediated H_2_O_2_ generation may be reduced in mycorrhizal-associated plants relative to non-colonized plants. This variation could be a mechanism by which mycorrhizae confer drought tolerance.

AMF can regulate plant physiological and molecular responses to tolerate drought stress, and they have a strong ability to cope with drought-induced oxidative damage via enhanced antioxidant defense systems (Zou et al., 2021). The ability of ROS scavenging ability in AMF-plant and NMF-plant were evaluated in this study. The ROS scavenging in plant maintained by both enzymatic and non-enzymatic antioxidants systems (Choudhary et al., 2018; Hasanuzzaman et al., 2020). The O_2_⋅^–^/H_2_O_2_ system that converts ROS into non-toxic molecules by enzymatic reactions is a key ROS scavenging pathway that may increase drought tolerance by mitigating ROS overaccumulation (Baxter et al., 2013; Mittler et al., 2022). SOD, an important catalyst in this pathway, was more abundant in mycorrhizal colonized plants than non-mycorrhizal competitors under drought conditions (Alscher et al., 2002). This result suggests that AMF inoculated seedlings may potentially have improved O_2_⋅^–^ scavenging ability, which could increase tolerance to drought stress. Similarly, the activity of CAT, GPX, MDAR, and DHAR significantly increased in AMF plants under drought stress in our study, which indicated that AMF plants had better H_2_O_2_ scavenging ability under drought stress by the higher activities of antioxidant enzyme. Boutasknit et al. (2021) also found that SOD, CAT, PX, and polyphenoloxidase significantly increased in AMF-associated plants under drought stress of young *Ceratonia siliqua* L. trees. Another, non-enzymatic antioxidants systems in plants, the ascorbic acid-glutathione (AsA-GSH) cycle, is utilized to detoxify H_2_O_2_ (Bashri and Prasad, 2016). In our study, when drought-stressed, the content of AsA, GSH and AsA/DHA, GSH/GSSG were higher in AMF-colonized roots compared with NMF-root, which indicates AMF-root could scavenge H_2_O_2_ more efficiently than NMF-root through non-enzymatic antioxidants systems. This is consistent with previous results: Langeroodi et al. (2020) showed that AMF (*R. irregularis*) colonization enhanced the activity of antioxidant defense systems (SOD, POD, AsA and GSH) in chicory and showed a commensurate H_2_O_2_ accumulation and reduced oxidative damage. Thereby, the enhancement of non-enzymatic antioxidants (AsA, GSH) and antioxidant enzymes (SOD, CAT, GPX, GR, APX, DHAR, and MDAR) in defense systems modify and guard against ROS burst in AMF plants (Bahadur et al., 2019; Zou et al., 2021). These results suggested that AMF plant may have better ROS scavenging ability than NMF plants, and are more capable of maintaining these antioxidant pathways under drought stress. Elevation of antioxidant defense systems in mycorrhizal plants under drought stress removes further ROS accumulation (Zou et al., 2021), thus mitigating toxicity effects on lipids, proteins and DNA, with significant consequences for plant functioning under stress.

In conclusion, AMF-inoculated *B. ceiba* had lower rates of O_2_⋅^–^ generation and lower H_2_O_2_ accumulation than other, non-colonized *B. ceiba*. AMF-inoculated *B. ceiba* seedlings also had upregulated levels of antioxidant enzymes (SOD, PX, CAT, APX, GPX, MDAR, and DHAR) and non-enzymatic antioxidants (AsA, GSH), compared with their non-mycorrhizal associated partners. When drought-stressed, these differences persisted, and AMF-colonized plants grew better than their uncolonized associates, suggesting that AMF-colonization may improve drought tolerance in *B. ceiba.*

## Data availability statement

The datasets presented in this study can be found in online repositories. The names of the repository/repositories and accession number(s) can be found in the article/Supplementary material.

## Author contributions

HC, ZL, and YZ conceived and designed the study. HC supervised this study and drafted the manuscript. ZL, CL, YG, and LH carried out the laboratory work and performed the analyses. ZL and YZ revised the manuscript. All authors read and approved the final manuscript.

## Footnotes

1
http://gigadb.org/dataset/100445

2
http://web.expasy.org/protparam/

3
https://www.genscript.com/wolf-psort.html

4
http://www.lynnon.com/

5
http://www.megasoftware.net/
