# Supplementary material for: Arbuscular mycorrhizal fungi contribute to reactive oxygen species homeostasis of Bombax ceiba L. under drought stress
Source: Front Microbiol. 2022 Sep 20;13:991781. doi: 10.3389/fmicb.2022.991781 (PMC9530913; doi:10.3389/fmicb.2022.991781)
Supplement: Supplementary file 1 [file Data_Sheet_1.docx]

Supplementary table 1 Primers used for quantitative reverse transcription (qRT)-PCR analysis.

| Genes | Forward Primer (5’- 3’) | Reverse Primer(5’- 3’) |
| --- | --- | --- |
| BcRbohA | TGGCCAGAGGTCTTGAGAAG | TCAAAACGCTTCTCCACAGC |
| BcRbohB | AGTGGTTCAGAGGGGTGATG | GCTTGAAGACCTTGTGCCAA |
| BcRbohC | CGTGGCGGTTCAAAGTGTAA | CATGCCCAACCTGCCTAATC |
| BcRbohD | AAGCAGGTTTCTCAGGAGCT | GCGGTGAAGAAGGCCATTAG |
| BcRbohDX1 | CCTTACTTTGGGGAGGAGCA | ACGCATTAAATCCGGTGAGC |
| BcRbohDX2 | TAGACGACTCTGTTGCGGTT | AGCTCCTGAGAAACCTGCTT |
| BcRbohE | TCGGTCCACTAAAGCAAGGT | GAATACGTCGGTCTTTGGCC |
| BcRbohF | ACGCATCTCAGACTCTAGCC | TCCACTTCCTCCCATGCATT |
| BcRbohFX1 | TACTCGTGGGCCTTGGAATT | TTGCTCCCTCGTTACCCAAT |
| BcRbohG | TGGGTGATGGCTCTTTGGAT | AGCAGTACCAGAGCCATGTT |
| BcRbohH | TCATGGCACTTCTTGGACCT | CCAAGGCCAGCAAATGATGT |
| BcRbohHX1 | GTCGCGGAACATGATTTCGA | AGGAAAATTTGGTGGTGGCC |
| BcRbohI | TGCGGCGATTGTTATTGGAG | GGCAGCTTAATCAGGTTCCG |
| BcRbohJ | AAGAAGGTGATGCTCGGTCA | CTAACGCCAATGGTCCACAG |
| Actin gene | TTTTGGCCAAGAATGCGGTT | ATGGACTGGGGCAACTGTAG |

Supplementary table 2 Homology matrix of 14 putative *Rboh* Nucleotide and Amino acid sequences from *B. ceiba*

| Nucleotide Identity | | | | | | | | | | | | | | | |
| --- | --- | --- | --- | --- | --- | --- | --- | --- | --- | --- | --- | --- | --- | --- | --- |
|  |  | BcRbohDX2 | BcRbohH | BcRbohC | BcRbohJ | BcRbohFX1 | BcRbohE | BcRbohI | BcRbohD | BcRbohHX1 | BcRbohB | BcRbohG | BcRbohF | BcRbohDX1 | BcRbohA |
| Amino  Acid  identity | BcRbohDX2 | | 70.90% | 57.40% | 88.50% | 66.40% | 54.50% | 70.70% | 57.70% | 55.30% | 58.30% | 55.60% | 89.10% | 54.40% | 71.00% |
|  | BcRbohH | 44.90% |  | 56.30% | 71.00% | 67.10% | 53.60% | 88.80% | 56.30% | 55.60% | 56.90% | 56.20% | 70.50% | 52.90% | 90.30% |
|  | BcRbohC | 72.90% | 44.70% |  | 58.00% | 58.80% | 56.10% | 57.20% | 91.50% | 59.80% | 92.00% | 56.10% | 57.60% | 55.20% | 56.70% |
|  | BcRbohJ | 48.90% | 42.50% | 45.40% |  | 65.90% | 54.00% | 70.10% | 57.90% | 55.80% | 58.30% | 54.80% | 87.50% | 54.00% | 70.50% |
|  | BcRbohFX1 | 55.70% | 48.40% | 54.40% | 49.70% |  | 55.90% | 66.70% | 58.70% | 55.40% | 59.10% | 55.80% | 65.60% | 54.80% | 66.30% |
|  | BcRbohE | 50.60% | 44.80% | 48.60% | 46.50% | 57.30% |  | 53.80% | 56.20% | 53.80% | 55.60% | 52.50% | 54.30% | 87.30% | 54.10% |
|  | BcRbohI | 54.90% | 48.00% | 53.10% | 48.20% | 92.00% | 56.70% |  | 57.50% | 55.90% | 57.90% | 55.80% | 70.20% | 53.70% | 89.10% |
|  | BcRbohD | 90.80% | 45.80% | 73.60% | 49.00% | 56.10% | 50.50% | 55.20% |  | 59.80% | 91.50% | 55.50% | 57.60% | 55.10% | 57.00% |
|  | BcRbohHX1 | 46.10% | 82.00% | 45.10% | 43.00% | 49.10% | 46.20% | 49.50% | 46.80% |  | 60.50% | 56.10% | 56.00% | 54.50% | 56.10% |
|  | BcRbohB | 69.60% | 47.60% | 67.10% | 47.90% | 56.80% | 50.70% | 55.60% | 70.30% | 47.70% |  | 56.20% | 58.00% | 54.90% | 57.30% |
|  | BcRbohG | 72.30% | 44.60% | 87.20% | 45.30% | 53.30% | 49.70% | 52.60% | 73.40% | 44.80% | 67.50% |  | 55.00% | 52.00% | 55.70% |
|  | BcRbohF | 54.50% | 47.30% | 52.80% | 49.80% | 92.90% | 56.90% | 89.60% | 55.10% | 48.40% | 55.60% | 52.40% |  | 54.40% | 70.30% |
|  | BcRbohDX1 | 90.70% | 45.70% | 73.10% | 49.20% | 56.10% | 50.40% | 55.10% | 92.50% | 46.40% | 70.50% | 72.90% | 55.00% |  | 53.80% |
|  | BcRbohA | 73.30% | 44.90% | 88.70% | 45.80% | 54.50% | 48.90% | 53.60% | 74.40% | 45.20% | 68.50% | 88.90% | 53.40% | 74.10% |  |

Supplementary table 3 Accession of sequences applied for polygenetic analysis

| Gene name | Species | Accession |
| --- | --- | --- |
| AtRbohA | *Arabidopsis thaliana* | gi\|166199750\| |
| AtRbohB | *A. thaliana* | gi\|166199748\| |
| AtRbohC | *A. thaliana* | gi\|166199746\| |
| AtRbohD | *A. thaliana* | gi\|166199745\| |
| AtRbohE | *A. thaliana* | gi\|75202468\| |
| AtRbohF | *A. thaliana* | gi\|75171015\| |
| AtRbohG | *A. thaliana* | gi\|75170840\| |
| AtRbohH | *A. thaliana* | gi\|75098431\| |
| AtRbohI | *A. thaliana* | gi\|18407959\| |
| AtRbohJ | *A. thaliana* | gi\|332196065\| |
| DzRbohAx1 | *Durio zibethinus* | XP_022736207.1 |
| DzRbohB | *D. zibethinus* | XP_022719422.1 |
| DzRbohC | *D. zibethinus* | XP_022776409.1 |
| DzRbohC1 | *D. zibethinus* | XP_022735959.1 |
| DzRbohD | *D. zibethinus* | XP_022768970.1 |
| DzRbohDx2 | *D. zibethinus* | XP_022772750.1 |
| DzRbohE | *D. zibethinus* | XP_022754767.1 |
| DzRbohE1 | *D. zibethinus* | XP_022751762.1 |
| DzRbohH | *D. zibethinus* | XP_022731096.1 |
| GhRbohA | *Gossypium hirsutum* | XP_040939505.1 |
| GhRbohA1 | *G. hirsutum* | XP_016739649.2 |
| GhRbohAx2 | *G. hirsutum* | XP_040963078.1 |
| GhRbohB | *G. hirsutum* | XP_016713704.2 |
| GhRbohC | *G. hirsutum* | XP_016726500.2 |
| GhRbohC | *G. hirsutum* | XP_016739842.2 |
| GhRbohC1 | *G. hirsutum* | XP_016736889.2 |
| GhRbohD1 | *G. hirsutum* | XP_016686984.1 |
| GhRbohD2 | *G. hirsutum* | XP_016752252.2 |
| GhRbohE | *G. hirsutum* | XP_016716911.2 |
| GhRbohH | *G. hirsutum* | XP_016720532.2 |
| GhRbohJ | *G. hirsutum* | XP_016745028.1 |
| GmRboHA | *Glycine max* | XP_003517484.1 |
| GmRboHAx1 | *G. max* | XP_006579505.1 |
| GmRboHB | *G. max* | XP_003554649.1 |
| GmRboHB1 | *G. max* | XP_003521697.1 |
| GmRboHC | *G. max* | XP_003526909.1 |
| GmRboHCx1 | *G. max* | XP_003522455.1 |
| GmRboHE | *G. max* | XP_006587062.1 |
| GmRboHHx1 | *G. max* | XP_006583585.1 |
| HsRbohAx1 | *Hibiscus syriacus* | XP_039036424.1 |
| HsRbohB | *H. syriacus* | XP_039029241.1 |
| HsRbohC | *H. syriacus* | XP_038993478.1 |
| HsRbohC1 | *H. syriacus* | XP_039069377.1 |
| HsRbohD | *H. syriacus* | XP_039009841.1 |
| HsRbohE | *H. syriacus* | XP_039054940.1 |
| HsRbohEx1 | *H. syriacus* | XP_039018551.1 |
| HsRbohX2 | *H. syriacus* | XP_039018552.1 |
| TcRbohA | *Theobroma cacao* | XP_007040366.2 |
| TcRbohA1 | *T. cacao* | XP_007037569.2 |
| TcRbohB | *T. cacao* | XP_007030987.2 |
| TcRbohC | *T. cacao* | XP_007038195.2 |
| TcRbohD | *T. cacao* | XP_017981756.1 |
| TcRbohE | *T. cacao* | XP_017983820.1 |
| TcRbohH | *T. cacao* | XP_007016604.2 |
