# Supplementary figures and images for: Arbuscular mycorrhizal fungi contribute to reactive oxygen species homeostasis of Bombax ceiba L. under drought stress
Source: Front Microbiol. 2022 Sep 20;13:991781. doi: 10.3389/fmicb.2022.991781 (PMC9530913; doi:10.3389/fmicb.2022.991781)

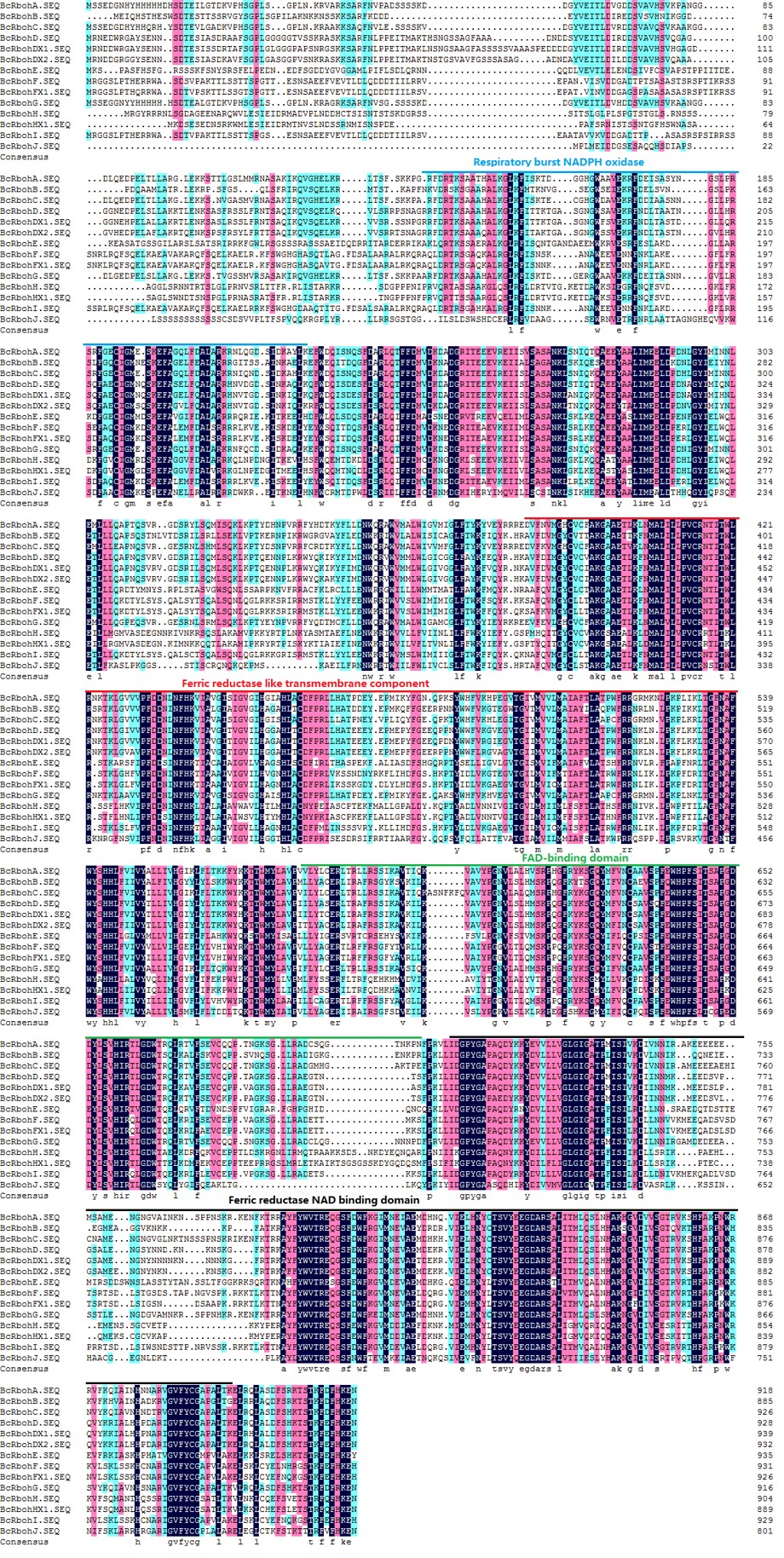

Supplement: Supplementary file 2 [file Image_1.JPEG]
